# Simulation and experimental data resemblance of darmstadt spark ignition engine with different turbulence models – A computational fluid dynamics cold flow data

**DOI:** 10.1016/j.dib.2022.108340

**Published:** 2022-06-02

**Authors:** A Gnana Sagaya Raj, Chandra Sekhar Mishra

**Affiliations:** Department of Mechanical Engineering, Sree Vidyanikethan Engineering College, Tirupati, Andhra Pradesh, India

**Keywords:** Darmstadt Engine, CFD, RNG k−ε, k−ω, Particle Image Velocimetry (PIV), Cold Flow, STAR-CD, TUD

## Abstract

The modelling of turbulence for IC engine applications is quite a challenging task. Large Eddy Simulation (LES) is the best approach to model the turbulence as the flow is three dimensional, chaotic, transient, diffusive, dissipative and intermittent. In this paper, a Computational Fluid Dynamics (CFD) data of in-cylinder air movement on TUD (Technische universitat Darmstadt) through Reynolds Average Navier-Stokes (RANS) approach with two different turbulence model, viz. Re-Normalized Group (RNG), K-Epsilon (*k*-ε) and K-Omega (*k*-ω) turbulence models for a single-cylinder, spark-ignition engine is analyzed. A commercial code STAR-CD (Solver for turbulent flow in arbitrary regions-Computational Dynamics) which works based on finite volume method is used for numerical analysis. Qualitative and quantitative data resemblance at a particular crank angle of interest throughout the inlet and compression stroke is analysed. CFD data was compared using the experimental data conducted on a single cylinder engine using a high speed Particle Image Velocimetry (PIV) technique, which was obtained from Darmstadt Technical University. Experimental data from the published literature were difficult to obtain and hence the above data is used for comparison. The resemblance data presented here are in terms of trapped mass of air, in-cylinder pressure, fluid flow pattern into the cylinder and the spatial variation of velocity at a particular interest of location and plane on the cylinder. The data offered in this work will be useful for academic researchers attempting to undertake computational fluid dynamics studies in diesel engines.

## Specifications Table

**Table:** 

Subject	Mechanical Engineering
Specific subject area	In-cylinder air flow measurement during suction and compression stroke in Darmstadt spark ignition engines
Type of data	Tables, figures
How data were acquired	Computer simulation
Data format	Raw, computed, analyzed
Description of data collection	The simulation is carried out in STAR CD – 4.22 version, which uses a polyhedral trimmed moving mesh This RANS simulations are conducted using a workstation with 12 cores and took about 3 days for RNG k−εand 7 days fork−ωturbulence model for 3 full cycle simulations. In this computation, the suction TDC starts at 360 CAD (Crank Angle Degree) and ends at 540 CAD. Compression starts when piston is at 540 CAD and ends at 720 CAD. The measured variation of pressure at the inlet with crank angle is used for this simulation. The boundary conditions, in terms of pressure 0.95 bar, are specified at the inlet of the intake port, which is located at about 10dp from the neck of the branch [1]. The simulated boundary condition data collected are in steps of 5 CAD from 0 CAD TO 720 CAD. A lengthy duct is included in the model so that the flow conditions at the intake port entrance are appropriate and do not need approximation. This also ensures the correct fluid mass with sensible turbulence level enters the cylinder as the flow evolves through the inlet duct. As stated in the literature [2] the pressure and temperature at the exit of the exhaust duct are specified to be 1.1 bar and 316.7 K respectively. The cylinder walls are specified to be isothermal at 400 K, the piston crown is at 500 K and the cylinder head is at 333 K. The turbulence conditions at the inlet are specified in terms of intensity as 5 % and length scale as 0.01 mm.
Data source location	Hopkinson Lab, Cambridge University Engineering Department, UK
Data accessibility	Repository name: Zenodo Repository and Mendeley DataData Identification number (permanent identifier, i.e. DOI number):10.17632/c3pgj4brhh.1Direct link to dataset: https://doi.org/10.5281/zenodo.6117269https://zenodo.org/record/6117269#.YmvBX9NBzIUhttps://data.mendeley.com/datasets/c3pgj4brhh/1

## Value of the Data

• This data can be used to predict the number of days needed to run the RANS simulation using two different turbulence model, viz. Re-Normalized Group (RNG), K-Epsilon (*k*-ε) and K-Omega (*k*-ω) turbulence models
• This dataset will be useful for academic researchers attempting to undertake computational fluid dynamics studies in diesel engines.
• This data is more valuable because in-cylinder air motion experimental setup is highly expensive to purchase. Hence, this data can be used as an initial and boundary conditions of CFD simulations.

## 1 Data Description

**Fig. 1:**
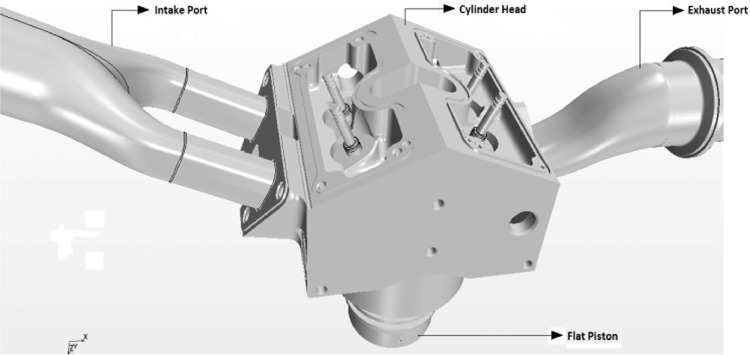
Assembled geometry of an optical engine.

**Fig. 2:**
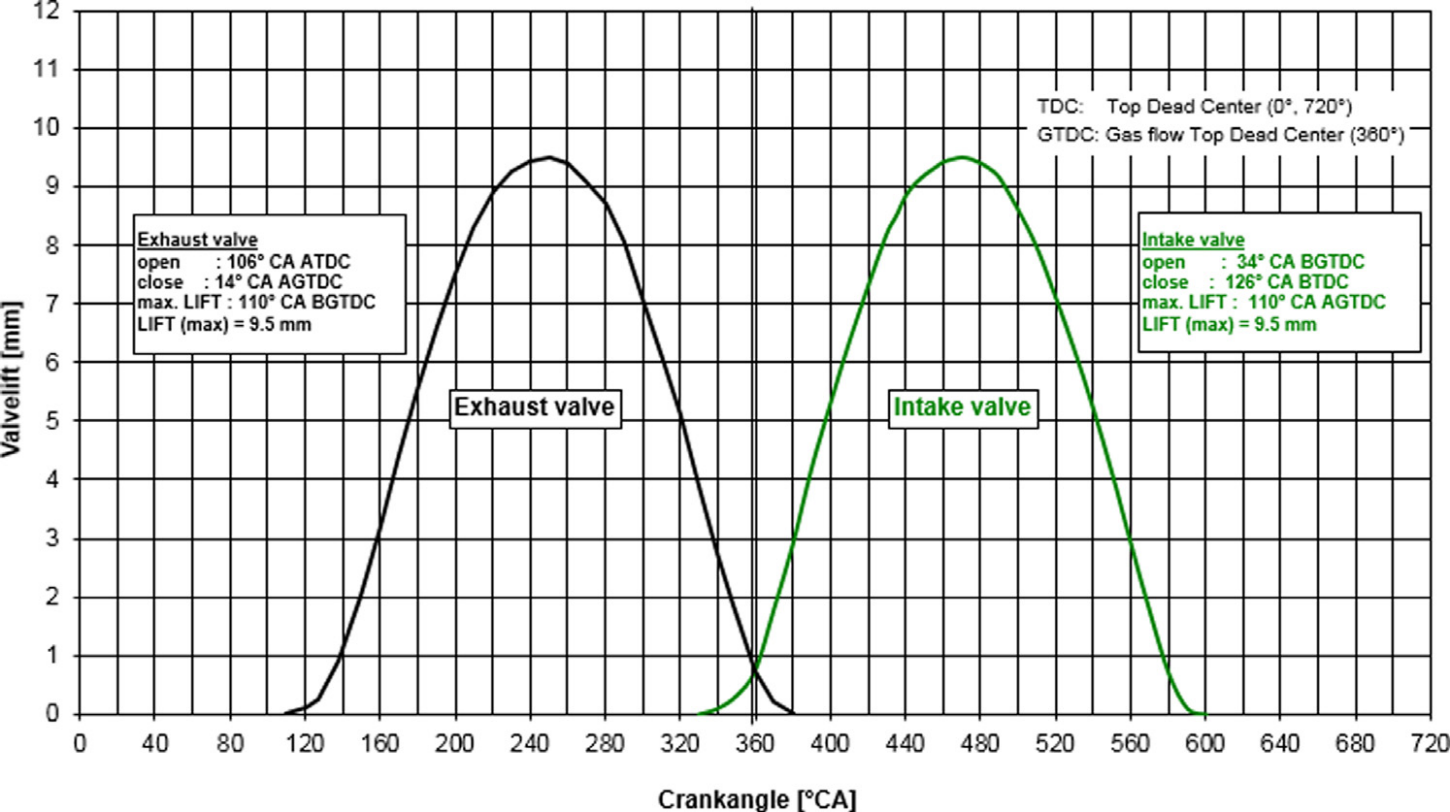
Intake and Exhaust Valve lift Data.

The investigated engine configuration (Table 1) is a realistic four stroke Spark Ignited (SI) engine with four poppet valve, pentroof cylinder head, flat piston and two intake and exhaust port which is shown in Fig. 1. Table 2 shows the simulated data of first cycle pressure boundary condition which is given as an input in the form of table format for second and third cycle simulation. The valve lift data is an important parameter to conduct the simulation which is available in Fig. 2.

**Table 1:** Engine Specifications.

Bore (mm)	86
Stroke (mm)	86
Clearance height (mm)	2.6
Connecting rod length (mm)	148
Engine Speed (rpm)	800
Compression Ratio	8.5
Opening of the Intake Valve (CAD)	34° before TDC
Closing of the Intake Valve (CAD)	126° before TDC
Opening of the Exhaust Valve (CAD)	106° after TDC
Closing of the Exhaust Valve (CAD)	14° after TDC
Intake/exhaust port diameter, d_p_ (mm)	60
Intake and exhaust port length (mm)	10d_p_ and 7d_p_

**Table 2:** Pressure Boundary conditions for cylinder, intake port and exhaust port at 800 rpm.

Crank Angle (deg)	Cylinder Pressure (bar)	Intake Port Pressure (bar)	Exhaust Port Pressure (bar)
0	0.970151	0.969624	0.993725
5	0.959293	0.974324	1.005232
10	0.952213	0.97542	1.019574
15	0.951394	0.96677	1.026317
20	0.945599	0.95739	1.025882
25	0.938701	0.948	1.019565
30	0.930997	0.937872	1.006435
35	0.922927	0.927945	0.99464
40	0.918865	0.917315	0.981614
45	0.913744	0.912456	0.977535
50	0.90858	0.914689	0.978911
55	0.91453	0.915478	0.98648
60	0.928675	0.919055	0.998589
65	0.932402	0.926476	1.009098
70	0.938016	0.938854	1.019594
75	0.943404	0.94852	1.021026
80	0.951166	0.9563	1.017983
85	0.957356	0.964388	1.009242
90	0.96232	0.97093	0.997603
95	0.965946	0.975154	0.988553
100	0.967433	0.974835	0.980189
105	0.965906	0.971099	0.979841
110	0.961899	0.967307	0.98407
115	0.95428	0.962744	0.992743
120	0.94754	0.957139	1.00265
125	0.944939	0.947944	1.01088
130	0.941679	0.940001	1.016261
135	0.936996	0.934929	1.014479
140	0.934192	0.931646	1.0093
145	0.931396	0.93042	0.999796
150	0.931556	0.929039	0.990345
155	0.933502	0.930381	0.983512
160	0.937024	0.934632	0.979594
165	0.941311	0.939416	0.982584
170	0.948013	0.94427	0.987694
175	0.954575	0.948316	0.996414
180	0.95827	0.953581	1.003948
185	0.959593	0.959494	1.00931
190	0.96153	0.963924	1.011706
195	0.962315	0.966006	1.008179
200	0.96436	0.964675	1.002476
205	0.964186	0.963351	0.993515
210	0.964144	0.961258	0.986829
215	0.965272	0.957387	0.982226
220	0.971521	0.95077	0.98148
225	0.989604	0.94104	0.985384
230	1.018094	0.929532	0.991278
235	1.055139	0.919066	0.998878
240	1.101079	0.912145	1.004298
245	1.156686	0.909552	1.007982
250	1.221618	0.908829	1.00761
255	1.297288	0.911879	1.002897
260	1.385282	0.919226	0.996859
265	1.491906	0.931129	0.989398
270	1.614744	0.944105	0.984574
275	1.761862	0.955916	0.98189
280	1.937976	0.966984	0.983539
285	2.146411	0.973904	0.98831
290	2.396755	0.975851	0.994098
295	2.70003	0.975624	1.000371
300	3.066154	0.972925	1.003648
305	3.511667	0.965834	1.004944
310	4.054714	0.956078	1.001871
315	4.716206	0.944311	0.996421
320	5.5156	0.933995	0.990343
325	6.466239	0.925894	0.984516
330	7.579296	0.920595	0.982186
335	8.830069	0.920345	0.981951
340	10.15734	0.92291	0.985795
345	11.44371	0.927358	0.990823
350	12.51641	0.934334	0.996462
355	13.17932	0.944339	1.000454
360	13.28634	0.954966	1.001296
365	12.80427	0.963806	1.000149
370	11.81696	0.970461	0.994751
375	10.52616	0.97358	0.990096
380	9.133344	0.972301	0.98483
385	7.796196	0.968481	0.98265
390	6.597275	0.962916	0.982659
395	5.575259	0.955779	0.98459
400	4.721285	0.94696	0.989594
405	4.021431	0.937687	0.994178
410	3.451115	0.930106	0.998351
415	2.986324	0.926246	0.999538
420	2.609484	0.924605	0.998405
425	2.298675	0.926921	0.995449
430	2.041464	0.932586	0.990642
435	1.829426	0.938913	0.986324
440	1.651855	0.946506	0.982861
445	1.506456	0.955211	0.982296
450	1.382262	0.96355	0.983781
455	1.280581	0.970544	0.987355
460	1.189165	0.97496	0.99179
465	1.116359	0.975759	0.995159
470	1.052157	0.973635	0.997251
475	0.998761	0.968641	0.99673
480	0.952361	0.961872	0.994898
485	0.913124	0.955059	0.991009
490	0.882754	0.947809	0.986594
495	0.86282	0.94047	0.979138
500	0.874316	0.934297	0.969045
505	0.90138	0.931316	0.959257
510	0.93439	0.931125	0.953281
515	0.963585	0.933609	0.958542
520	0.985861	0.938959	0.974738
525	1.005051	0.945729	0.996309
530	1.02187	0.952461	1.014124
535	1.038552	0.959339	1.024442
540	1.049776	0.965609	1.026184
545	1.048564	0.970853	1.020409
550	1.033936	0.974214	1.012427
555	1.009132	0.974084	1.001647
560	0.984684	0.971431	0.987686
565	0.966496	0.966687	0.97171
570	0.957121	0.960075	0.958451
575	0.950772	0.953224	0.953481
580	0.948725	0.947183	0.957701
585	0.955838	0.941685	0.968388
590	0.972968	0.937198	0.981035
595	1.000969	0.935074	0.992768
600	1.023572	0.93551	1.005992
605	1.036381	0.938676	1.019606
610	1.037904	0.943911	1.027964
615	1.033237	0.950471	1.027483
620	1.026991	0.957519	1.017499
625	1.0145	0.963629	1.002667
630	0.994646	0.968808	0.98882
635	0.969398	0.972596	0.977335
640	0.955335	0.975025	0.968611
645	0.953993	0.974875	0.965055
650	0.963946	0.971862	0.968198
655	0.976486	0.967185	0.9798
660	0.98952	0.961291	0.996495
665	1.006915	0.954915	1.011511
670	1.024251	0.949543	1.021701
675	1.029674	0.945533	1.026677
680	1.02117	0.942605	1.023176
685	1.006879	0.94164	1.011945
690	0.990485	0.942337	0.997309
695	0.97587	0.945283	0.982127
700	0.962374	0.95025	0.971121
705	0.953315	0.95571	0.967149
710	0.95886	0.961395	0.970448
715	0.96744	0.966176	0.980549
720	0.97081	0.969155	0.992546

**Fig. 3:**
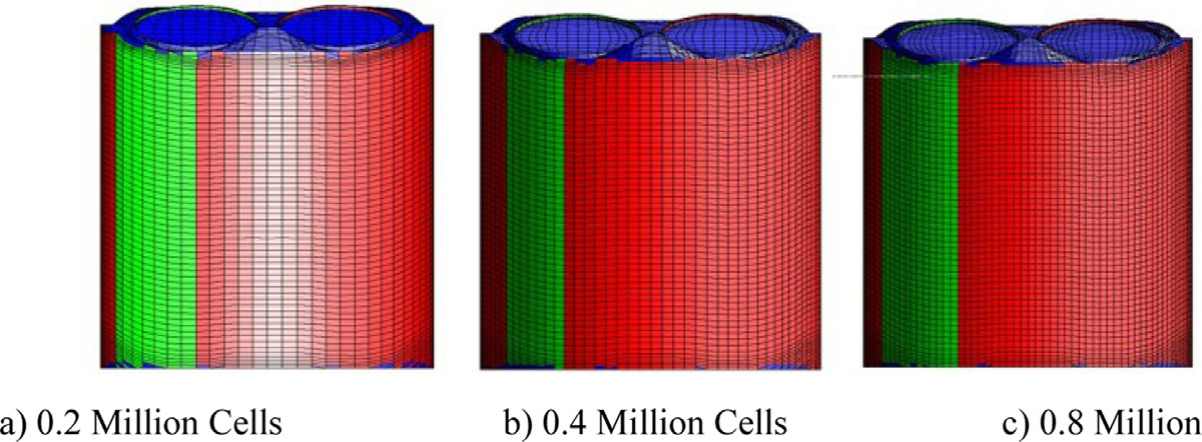
Variation of Mesh Sizes from 0.2 to 0.8 million cells.

The good quality of moving mesh generation in STAR-CD to perform an engine simulation is quite a challenging task. In this study, a new automatic mesh generation method was used to generate a three different polyhedral trimmed mesh sizes. When the piston is at BDC, the created mesh comprises of 0.2, 0.4, and 0.8 million cells (Fig. 3) in this study, and this grid independence study was carried out by applying RNG k−εturbulence model to figure out the best mesh size and to acquire a higher accuracy of findings when compared to experimental data.

**Fig. 4:**
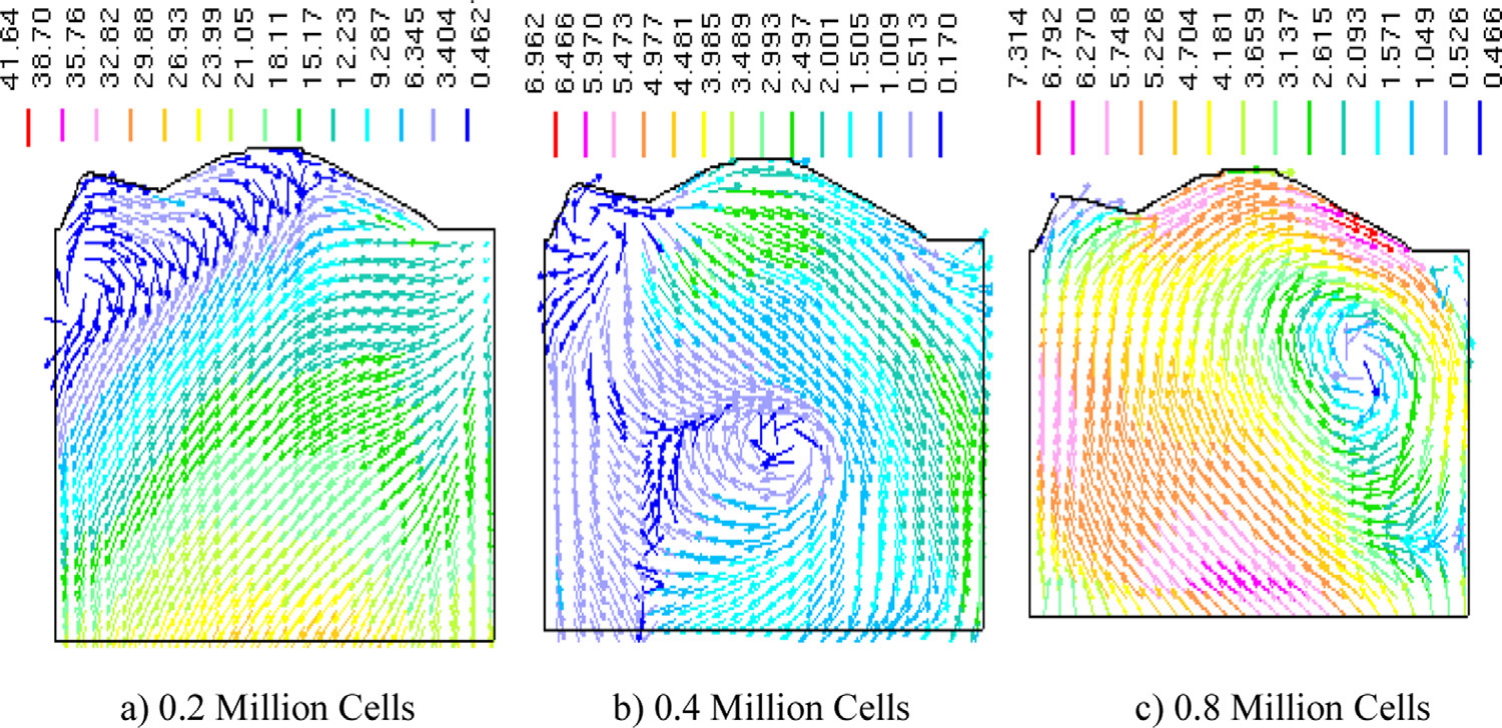
Resemblance of in-cylinder velocity vector data for three different mesh sizes during compression stroke @630 CAD.

Fig. 4a-c depicts a resemblance data of the in-cylinder flow velocity pattern for three different mesh sizes at the cylinder's centre plane. Visual study of Fig. 4b and c shows that there is a clockwise (CW) vortex structure inside the cylinder for 0.4 and 0.8 million cells, but it is absent for 0.2 million cells (Fig. 4a). For 0.4 million mesh, the CW vortex is in the middle of the cylinder, whereas for 0.8 million mesh, it is on the right hand side of the cylinder block. Another important observation is that the 0.4 and 0.8 million mesh size are predicting almost the same in-cylinder velocity of 6.9 and 7.3 m/s.

**Fig. 5:**
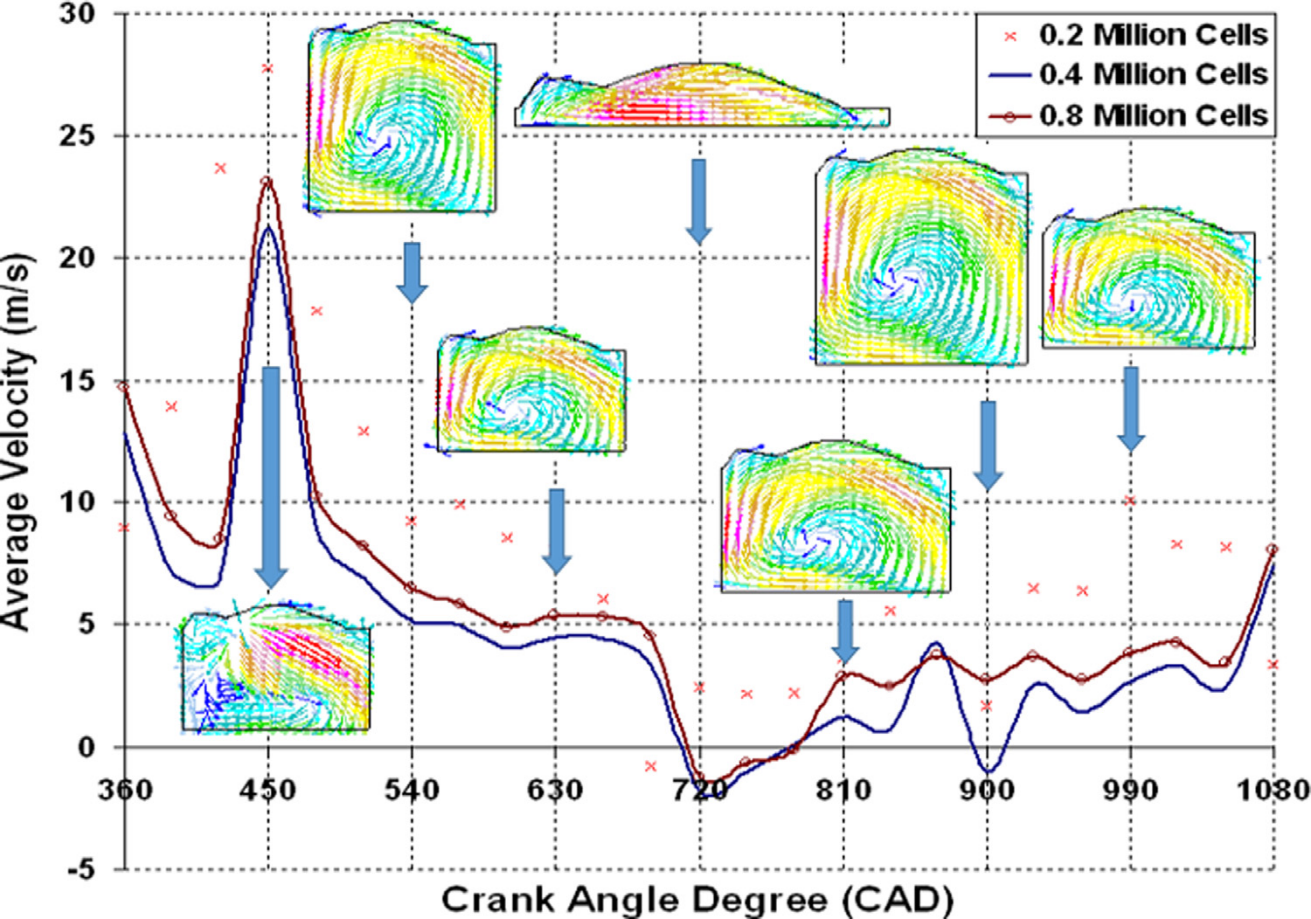
Data variation of average velocity Vs CAD.

**Fig. 6:**
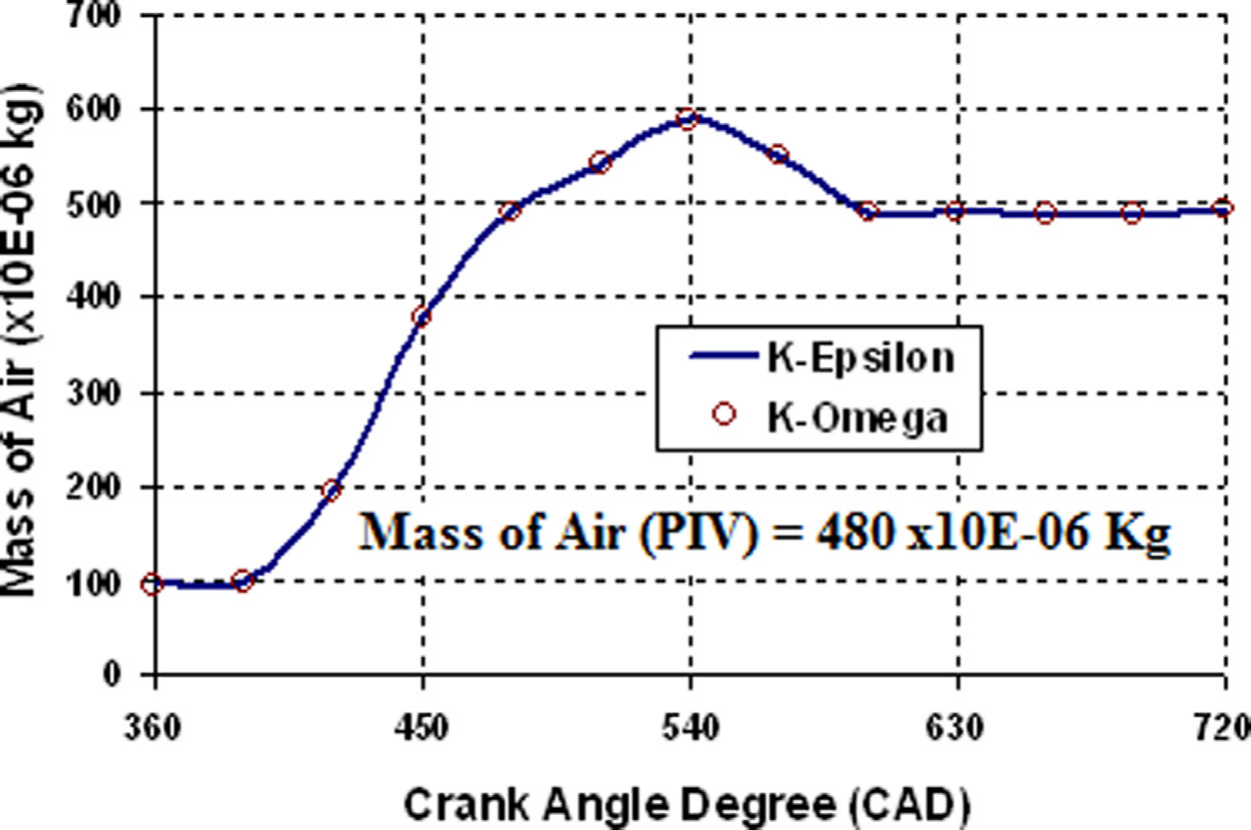
Resemblance data of mass of air Vs CAD.

**Fig. 7:**
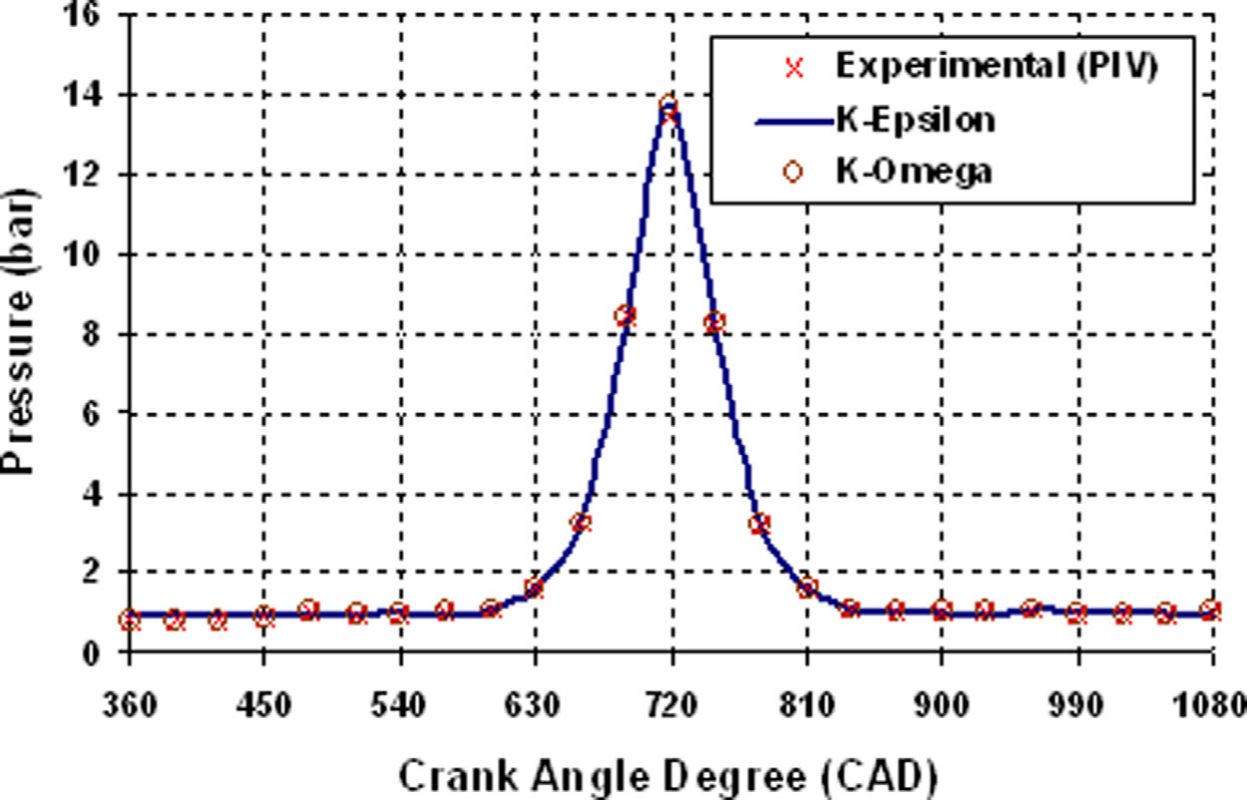
Resemblance data of in-cylinder pressure Vs CAD.

Fig. 5 depicts the data variation of average velocity superimposed over an in-cylinder flow pattern at various crank angle degrees in the cylinder's mid-plane. Based on this graph, it is clear that 0.4 and 0.8 million cells exhibit a similar pattern with a 7 percent error margin. Fig. 6 shows the resemblance data of mass of air versus CAD. The PIV experiments' recorded pressure versus time dependent boundary conditions are used as computational input to confirm that the in-cylinder mass of air and pressure standards match the experimental findings. Fig. 7 shows the resemblance of in-cylinder pressure versus CAD. From Fig. 6 and Fig. 7, it is clearly seen that RNGk−εand k−ωturbulence models find the similar trapped in-cylinder mass of air as 480 × 10E-06 kg after the intake valve closes at 126 CAD bTDC and an in-cylinder peak pressure as 13 bar at compression TDC (at 720 CAD) as compared to experimental results.

**Fig. 8:**
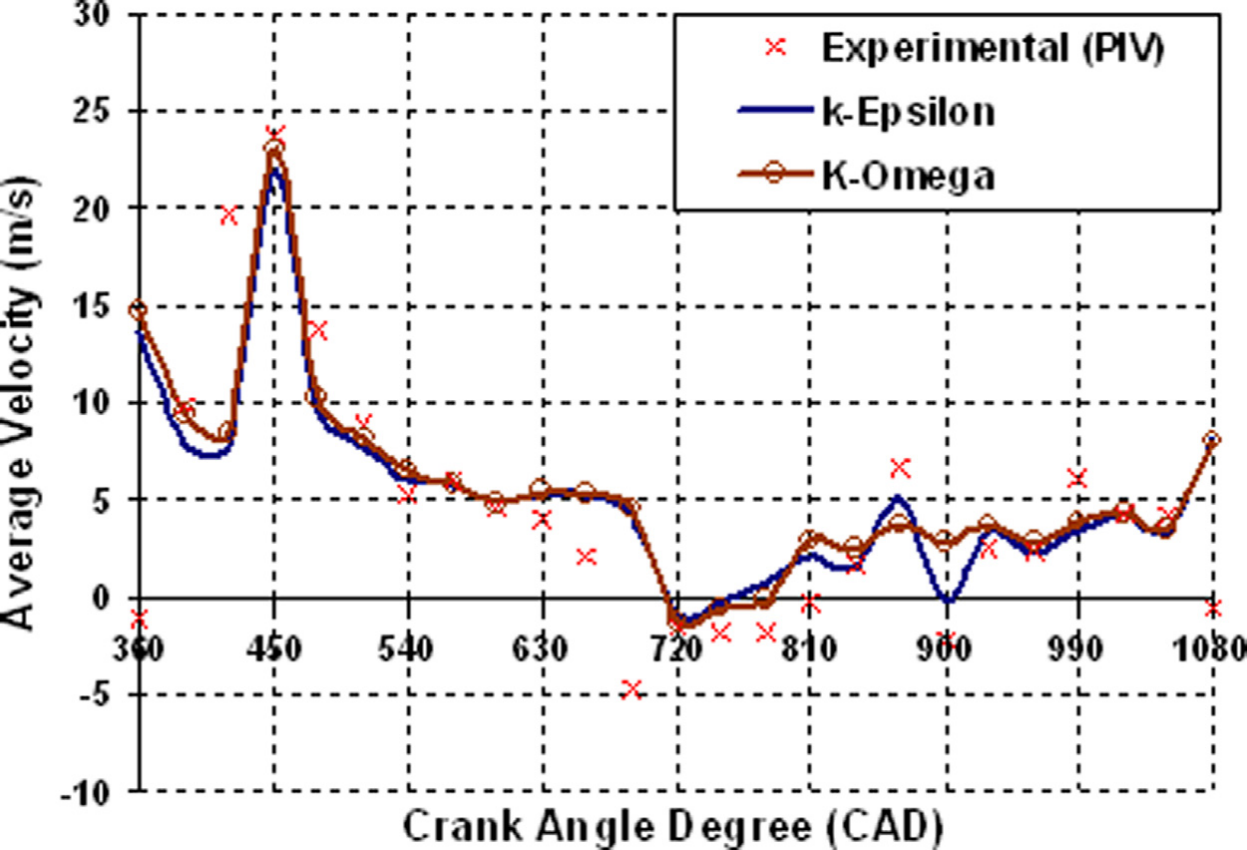
Resemblance data of average velocity Vs CAD.

Fig. 8 illustrates the comparison of experimental and computed average velocity data of various crank angle degrees from intake to exhaust stroke within the combustion chamber. The graph clearly shows that the maximum velocity is reached at 450 CAD and steadily drops until the end of the compression stroke. The graphical figure also shows that the RNG k−ε turbulence model under-predicts at the start of the intake stroke and at the end of the expansion stroke. However, k−ω the turbulence model agrees well with observed average velocity and predicts with more accuracy than the K-epsilon turbulence model.

**Fig. 9:**
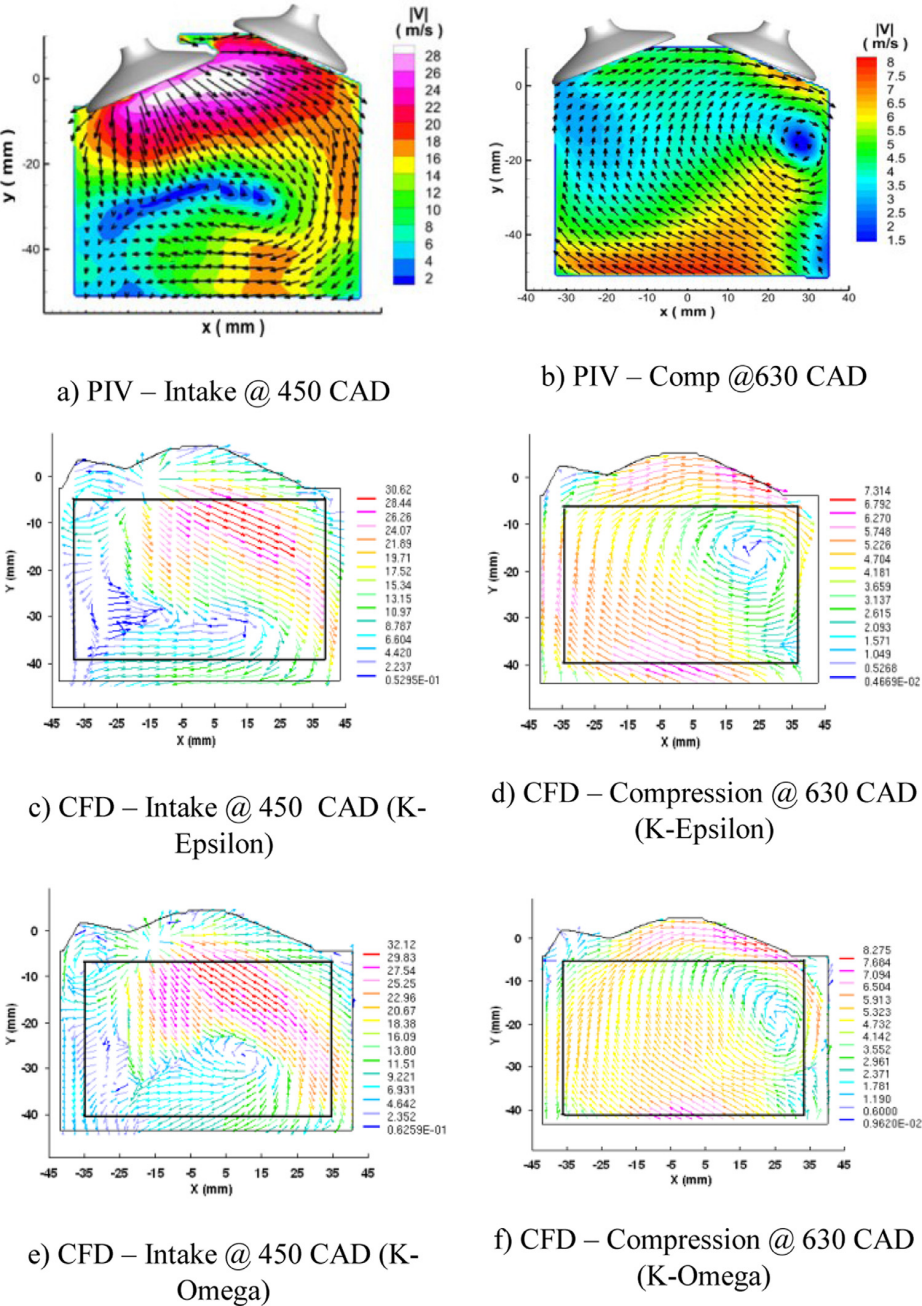
Resemblance data of Experimental and Computational in-cylinder Velocity during intake and compression stroke.

Fig. 9 shows the resemblance of experimental and computational in-cylinder Velocity during intake and compression stroke. The left column of the figure explains the in-cylinder flow pattern during the suction stroke at 450 CAD using RNGk−εand k−ω turbulence model. The right column of the figure illustrates the in-cylinder flow characteristics during the compression stroke at 630 CAD using the above mentioned turbulence models.

**Fig. 10:**
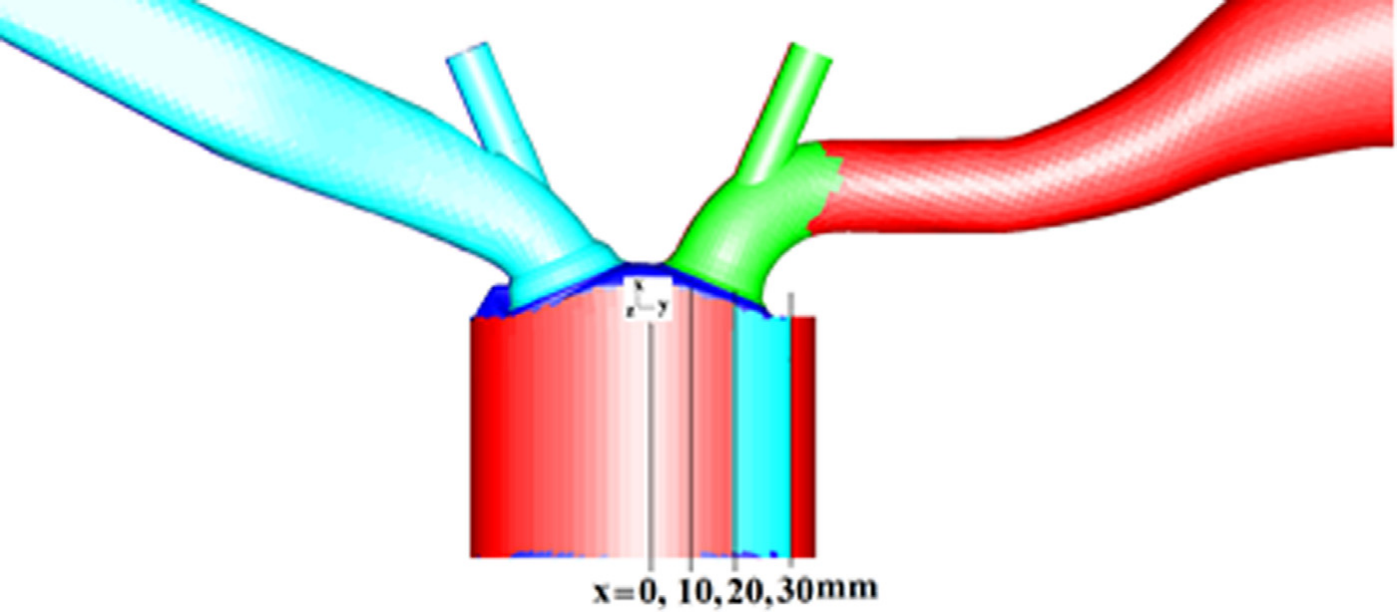
Position of Measurements on cylinder plane.

Fig. 10 shows the position of measurements on cylinder plane. The RANS approach computational analyses are further extended to study the average velocity, comparison of flow structure and spatial velocities at different locations of x = 0, 10, 20 and 30 mm during the intake and compression stroke and compared with an experimental results available from the TUD engine geometry.

**Fig. 11:**
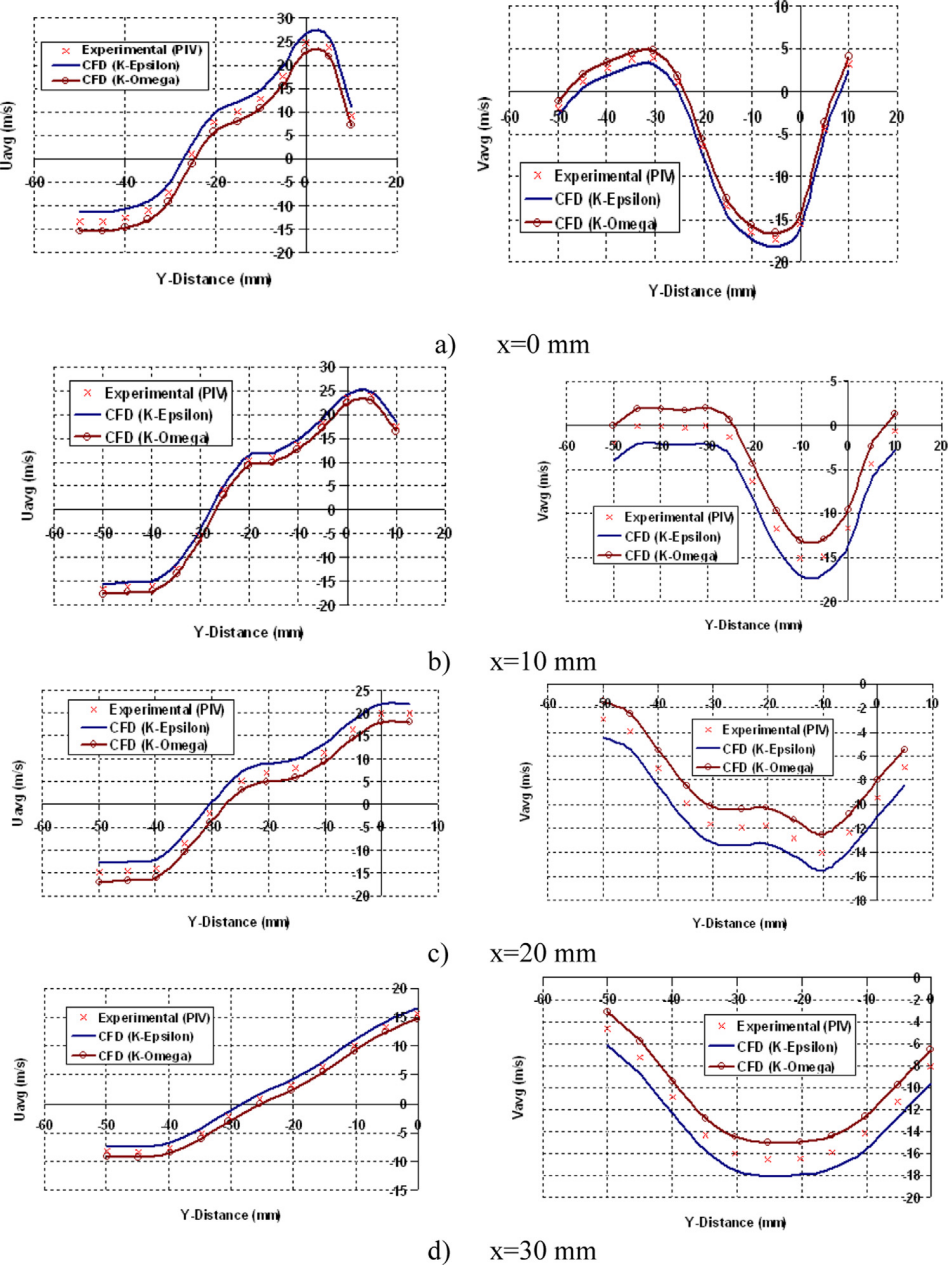
Resemblance data of Spatial Velocity during Intake Stroke at 450 CAD.

Fig. 11 depicts the resemblance of Spatial Velocity during Intake Stroke at 450 CAD. The figure clearly shows that the flow directions are in upward direction for positive velocity and downwards for negative velocity. The expected findings of the RNG k−ε and k−ωturbulence models accord well with the data from the PIV experiment.

**Fig. 12:**
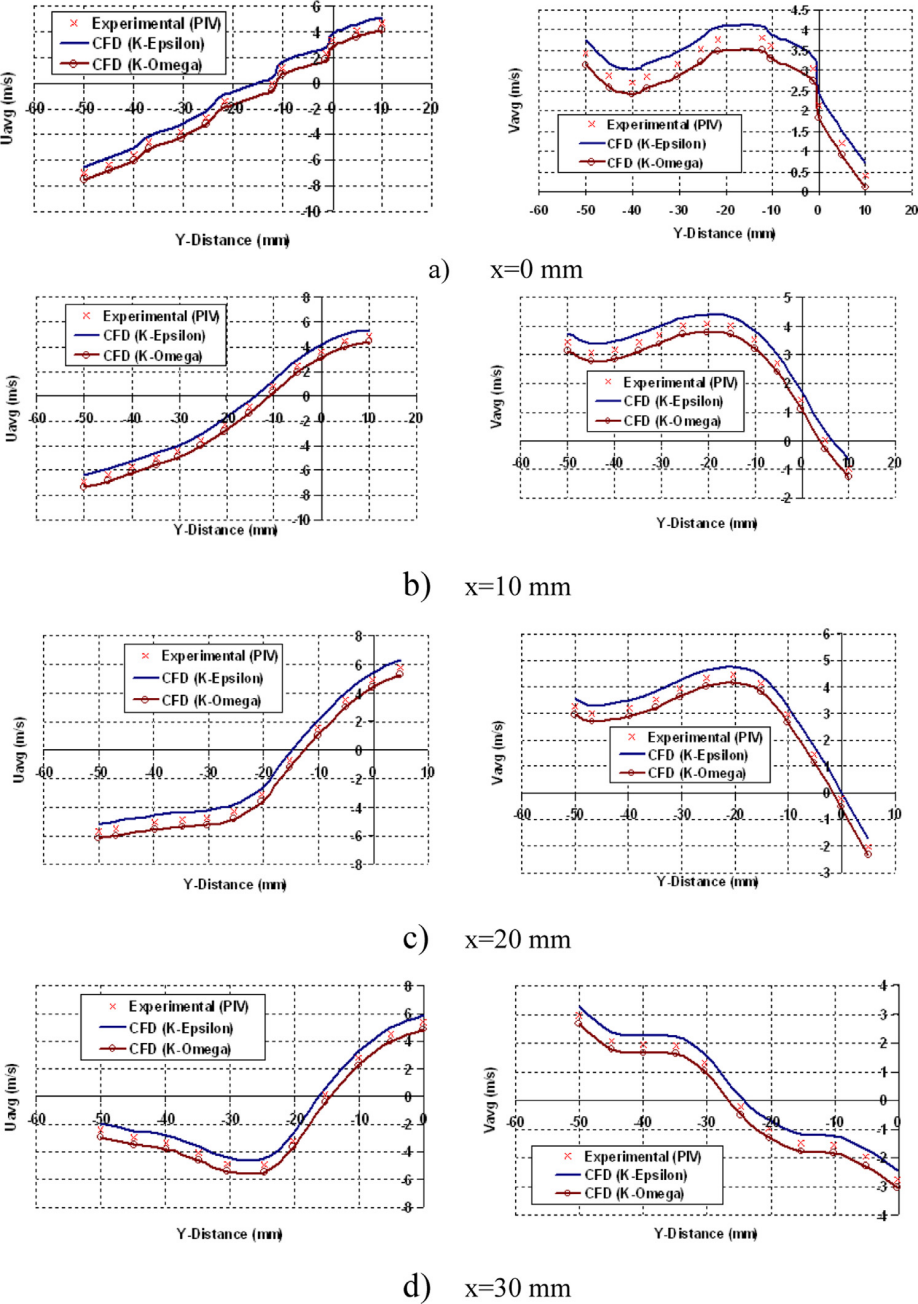
Resemblance data of Spatial Velocity during Compression Stroke at 630 CAD.

Fig. 12 shows the resemblance of Spatial Velocity during Compression Stroke at 630 CAD. From Fig. 12, it is seen for both U_avg_ and V_avg_, RNGk−εturbulence model over predicts and k−ωturbulence model under predicts as compared to measured data. The computational results, on the other hand, are in good agreement with the investigative data.

## 2 Experimental Design, Materials and Methods

The computational data of the TUD optical SIDI (spark ignited direct injection) engine geometry are compared with the experimental data of TUD geometry [2]. The experiments were carried out on a single cylinder, spark-ignition engine with an overhead valve pentroof cylinder head and motored at 800 rpm, using an HS-PIV (High speed particle image velocimetry) technique to visualize the in-cylinder velocity and vortex structure during the intake (450 CAD) and compression stroke (630 CAD). The cylinder liner was raised to a height and thickness of 55 × 20 mm in the detailed exploration of PIV technology by installing a transparent quartz glass liner for an optical access of the fire deck combustion chamber and it works as an interrogation window to view the flow [1,2]. As a result, during the post-processing of computational findings to validate the data [3], [4], [5], [6], [7], [8], [9], the identical 55 mm cut piece from the cylinder head is used. The four positions of planes are taken for measurements. The measurements planes in experiment are in radial direction and similarly positioned from the cylinder central axis. The four measurement position of planes are at x = 0, 10, 20, 30 mm, which are selected from the available experimental data. Same positions are chosen in CFD study to validate the data.

### 2.1 Computational details

The fluid domain is obtained from the complete built engine block in such a way that the entire surface is closed and there are no duplicate surfaces. The separated engine block surfaces are the cylinder, pentroof head, flat piston, intake and exhaust valves, and intake and exhaust ports. The surfaces are next cleaned with STAR CCM+ 9.06 to ensure that there are no mistakes of free edges, repeated edges, sharp pair of angles, self-intersection, or shell orientation on cells. After cleaning these surfaces, they are assigned as separate components with various shells, as. Finally, it is surface meshed in STAR CCM+ and imported into es-ice for volume mesh generation and moving mesh generation.

In this CFD simulation, an engine is run at 800 rpm with a constant compression ratio of 8.5. The simulation is run for three cycles, with the first cycle findings ignored due to the initial transients, and only minor variations noticed between the second and third cycles. Therefore, only the second cycle results are used after attaining the stabilized results. This RANS simulation took roughly 3 days for RNG k−ε and 7 days for k−ω turbulence model for three complete cycle runs on a machine with 12 cores. The convective fluxes present in the momentum equations are solved using the monotonic advection reconstruction scheme (MARS), and the Arbitrary Langrangian-Eulerian (ALE) approach is used to discretize the governing equations involved in moving mesh. The pressure coupled velocity equations were solved using the PISO technique.

### 2.2 Governing equation

The momentum and mass equations solved in Cartesian tensor form for the unsteady, incompressible/compressible, three-dimensional in-cylinder flow [5] are represented as

(1) $$\frac{\partial \rho }{\partial t}+\frac{\partial }{\partial x_{j}}\left(\rho u_{j}\right)=s_{m}$$

(2) $$\frac{\partial (\rho u_{i})}{\partial t}+\frac{\partial }{\partial x_{j}}\left(\rho u_{j}u_{i}-\tau_{ij}\right)=-\frac{\partial p}{\partial x_{i}}+s_{i}$$

where, t – time, x_j_ - Cartesian coordinate (*j* = 1, 2, 3), u_i_ - Absolute fluid velocity component in x-direction, piezometric pressure $=p_{s}-\rho_{0}g_{m}x_{m}$ where $p_{s}$ is static pressure, ρ_0_ is reference density, g_m_ are gravitational acceleration components and x_m_ are coordinates relative to a datum where ρ_0_ is defined as Density, ρ

$$=\left(\frac{p}{RT\sum_{M}\frac{m_{m}}{M_{m}}}\right)$$

where m_m_ - mass fraction of a constituent with molecular weight *M_m_, T –* temperature, R - universal gas constant, τ_ij_- stress tensor components, S_ij_-mass source, S_i_-momentum source components (assumed to be negligible). Assuming Newtonian flow, the following constitutive relation is stated to relate the components of the stress tensor $\tau_{ij}$ to the velocity gradients:

(3) $$\tau_{ij}=2\mu s_{ij}-\frac{2}{3}\mu \frac{\partial u_{k}}{\partial x_{k}}\delta_{ij}-\bar{\rho }\bar{{u^{\prime }}_{i}{u^{\prime }}_{j}}$$

Where µ- molecular dynamic fluid viscosity, δ_ij_ - the Kronecker delta (= 1 when i = j, and 0 otherwise)

(4) $$s_{ij}=\frac{1}{2}\left(\frac{\partial u_{i}}{\partial x_{j}}+\frac{\partial u_{j}}{\partial x_{i}}\right)$$

The fluctuations about the ensemble average velocity are represented by the rightmost component in Eq. (3), which reflects the increased Reynolds stresses owing to turbulent motion. Through turbulent models, the Reynolds stresses are related to the mean velocity fields.

## CRediT authorship contribution statement

**A Gnana Sagaya Raj:** Conceptualization, Methodology, Software, Validation, Visualization, Supervision, Investigation, Writing – original draft, Writing – review & editing. **Chandra Sekhar Mishra:** Writing – review & editing.

## Declaration of Competing Interest

The authors declare that they have no known competing financial interests or personal relationships that could have appeared to influence the work reported in this paper.

## Data Availability

TUD Boundary Condition (Original data) (Boundary Condition).
